# Cohorts and community: a case study of community engagement in the establishment of a health and demographic surveillance site in Malaysia

**DOI:** 10.3402/gha.v7.23176

**Published:** 2014-05-06

**Authors:** Pascale Allotey, Daniel D. Reidpath, Nirmala Devarajan, Kanason Rajagobal, Shajahan Yasin, Dharmalingam Arunachalam, Johanna Debora Imelda, Ireneous Soyiri, Tamzyn Davey, Nowrozy Jahan, The Seaco Team

**Affiliations:** 1 South East Asia Community Observatory (SEACO), School of Medicine and Health Sciences, Monash University, Segamat, Malaysia; 2 Global Public Health, Jeffrey Cheah School of Medicine and Health Sciences, Monash University, Malaysia; 3 Centre for Population and Urban Research, School of Political and Social Inquiry, Faculty of Arts, Monash University, Clayton, Australia; 4 Post Doc Fellowship, Amsterdam Institute for Social Science Research, University of Amsterdam, Amsterdam, The Netherlands; 5 Department of Social Welfare, Faculty of Social and Political Science, University of Indonesia, Depok, Indonesia

**Keywords:** community engagement, dynamic cohort, HDSS, cohort recruitment, cohort retention

## Abstract

**Background:**

Community engagement is an increasingly important requirement of public health research and plays an important role in the informed consent and recruitment process. However, there is very little guidance about how it should be done, the indicators for assessing effectiveness of the community engagement process and the impact it has on recruitment, retention, and ultimately on the quality of the data collected as part of longitudinal cohort studies.

**Methods:**

An instrumental case study approach, with data from field notes, policy documents, unstructured interviews, and focus group discussions with key community stakeholders and informants, was used to explore systematically the implementation and outcomes of the community engagement strategy for recruitment of an entire community into a demographic and health surveillance site in Malaysia.

**Results:**

For a dynamic cohort, community engagement needs to be an ongoing process. The community engagement process has likely helped to facilitate the current response rate of 85% in the research communities. The case study highlights the importance of systematic documentation of the community engagement process to ensure an understanding of the effects of the research on recruitment and the community.

**Conclusions:**

A critical lesson from the case study data is the importance of relationships in the recruitment process for large population-based studies, and the need for ongoing documentation and analysis of the impact of cumulative interactions between research and community engagement.

Health and demographic surveillance systems (HDSS) are dynamic or open cohorts based on a regular, longitudinal surveillance of the entire population within a defined geographic location. Subject to consent, all residents are enrolled, and sequentially, all new immigrants and births to the designated area are recruited into the cohort during periodic updates of the census. All deaths and emigrants from the area are attritions from the cohort. Census updates are conducted on 6- to 24-month cycles depending on the size of the area covered, resources available, and the purpose of the HDSS, to ensure an accurate denominator at any point in time (1, 2). Typically, a core set of demographic and health status questions are repeated at each census update, capturing information that includes disease prevalence, risk factors, health-related behavior, and social transitions; building a rich longitudinal data base which embeds a dynamic and temporal understanding of individual health and wellbeing within the context of family, community, and environment. Historically, HDSS sites were established in resource-poor settings in Africa and Asia where vital registration systems were ineffective and unreliable. The HDSS data provided an alternative source of evidence to glean some understanding of disease trends and to undertake community-based, disease-specific intervention studies (2, 3). Many of the extant HDSS sites have come together under the umbrella of an International Network of field sites with continuous Demographic Evaluation of Populations and Their Health (INDEPTH). INDEPTH represents 42 HDSS sites from countries in Africa, Asia, and Oceania (2).

In November 2011 a new HDSS, the South East Asia Community Observatory (SEACO), was launched in Segamat district, Johor, Malaysia. The SEACO site is atypical of the INDEPTH Network sites because Malaysia already has a very good universal population registration system and therefore the motivation was neither to address the ‘intractable lack of population based data on health’ (p. 579), nor to address a specific research question (2). SEACO was established as a generic ‘community health laboratory’; a multi-purpose research platform to enable a broad range of life course research projects and complex interventions to be undertaken in both health- and non-health-related disciplines. SEACO's vision is to develop a research and training site providing high-quality infrastructure for conducting community-based whole of life research. SEACO aims to achieve this through the collection and sharing of high-quality data and methods, and protection of, and service to the communities involved with SEACO.

A significant hurdle in the initial phase of the SEACO establishment was in the recruitment of the population, to ensure as high a response rate as possible. Participation in non-commercial, scientific research is generally voluntary and therefore, unsurprisingly, recruitment is a challenge. A significant number of study protocols are abandoned as a result of the inability of researchers to recruit sufficient numbers of participants to power the study (4). A study of controlled trials that had been published in the *Lancet* and *British Medical Journal* reported that approximately 60% of researchers were unable to meet their recruitment targets within the proposed time frame (5). Furthermore, once recruited, the retention of participants toward longitudinal follow-up is also a challenge (6–8). Strategies to improve recruitment have been explored through a number of studies and can be categorized under two broad headings: those that appeal to participants’ sense of being a part of and contributing to knowledge and society; and those that provide monetary and other tangible incentives. The former include invitation letters from eminent members of society or respected institutions, or opportunities for direct engagement with researchers through telephone calls or face to face meetings. The latter include reimbursement for lost opportunity and direct costs associated with participation, tokens of appreciation, or entry into lotteries. Other strategies include reducing the effort required for participation through simplified consent processes and the use of opt-out rather than opt-in invitations (9, 10). Reviews to synthesize the evidence on the effectiveness of various approaches to recruitment and retention in longitudinal studies (4, 11) are inconclusive and have highlighted the difficulty in identifying the best practice because researchers often use multiple approaches. In addition, recruitment strategies and challenges are often not well described in publications and research reports. Furthermore the evaluation of recruitment approaches is usually not built into the methodology of studies.

The experiences from other HDSS sites did not provide much direction for research recruitment. Many of the other sites are affiliated with or run through national ministries of health and disease control programs. The recruitment for research is therefore often conflated by their multiple roles in routine, statutory disease surveillance and public health interventions, both of which fulfill concrete functions associated with government that supersede research (12). Participants are therefore not responding solely to an invitation to a research program (13, 14). In sites where this is not the case, the experience of recruitment is poorly documented.

The challenge for SEACO was two-fold. The first was to gain consent for recruitment from the entire population within the geographical area designated for the site. The second was to obtain consent from participants to be retained in a program of research that, by design, did not have specific and clearly articulated research outcomes or an end point. Essentially, we were asking members of the community to sign on for an open-ended program of research that would require regular, though infrequent, visits from research staff on a range of areas of research (many of which are not yet determined) that related to the broad area of health and well-being. This placed a significant onus of accountability on the research team to ensure that no promises of specific outcomes were made to the community that were outside the remit or responsibility of the research team to provide – and that the nature of the HDSS design was made clear to participants.

We envisaged a model of working with the community toward a shared ownership of SEACO, providing a vehicle through which research undertaken through SEACO would evolve from investigator-driven questions to joint- and community-directed research priorities. We also expected that the partnership with the community would enhance the accuracy of responses and quality of the data. The strategy adopted by SEACO was therefore a highly consultative process of community engagement and partnership building, designed to enhance recruitment and retention of the target population through activities that would inform, consult, involve, and empower the community in the district (15). Our community engagement therefore had to be designed and assessed both as an ongoing process for developing a working relationship and with an outcome of high rates of recruitment and retention.

It is important to note, that in spite of increasing ethical requirements for community engagement, there is very little guidance on how it is done and how to assess the quality of the process and the outcomes of community engagement (14, 16). We therefore chose a case study approach to explore systematically the implementation and outcomes of the community engagement strategy. The research was important to provide a rigorous assessment of a real-life process and to understand its effects on the research that will be undertaken through SEACO. In this paper, we provide a detailed description and analysis of the community engagement strategy for recruitment of the population for the SEACO HDSS and discuss the broader implications of community engagement for the quality of the research and for the recruitment and maintenance of cohorts.

## The setting – Segamat, Johor

**Fig. 1:**
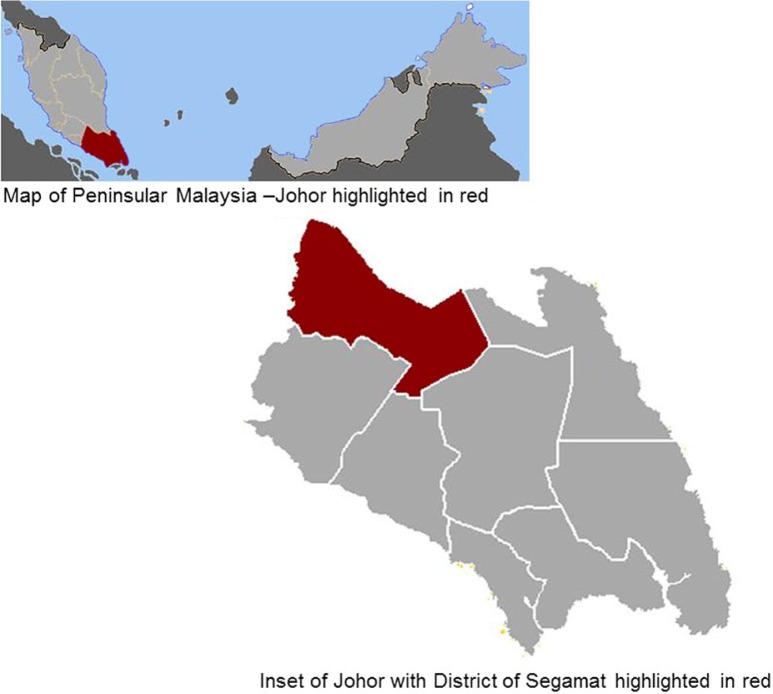
Map of SEACO site location.

SEACO is located in Segamat, the northernmost district in the southern state of Johor, Peninsular Malaysia (see Fig. 1). It is largely semi-rural, although there are a number of remote rural communities. The economic base of the district is predominantly agricultural with extensive oil palm and rubber plantations and it is well known for its durian orchards. According to the last national census (2010), the total population of Segamat is approximately 170,000 with 50% *Bumiputra* (‘son of the earth’ describing the combination of ethnic Malay and Indigenous Orang Asli people), 36% ethnic Chinese, 9% ethnic Indian with the remaining accounting for non-citizens. Foreign workers from countries like Bangladesh, Indonesia, and Nepal travel to rural areas of Malaysia to work on the plantations (17). This profile approximates the ethnic mix of the national population.

Health care is provided predominantly through a well-established government primary health care service with 10 health clinics (*Klinik Kesihatan –* KK) and 25 community clinics (*Klinik Desa* – KD) and a 300+ bed district hospital. The health system meets the national benchmark to provide one KK and four KDs per 20,000 people. There is also a growing private health care sector made up of private GP practices and a popular complementary and alternative medicines sector.

The SEACO site covers 5 of the 11 subdistricts or *Mukim* in Segamat. These are Sungai Segamat – the district capital, Bekok – on the eastern border and the most remote and home to the Orang Asli community, Chaah which is predominantly plantations, Jabi and Gemereh. These Mukim were selected to provide the diversity of rural and semi-rural activity, ethnicity, and geographical variation. Despite the promotion of its multi-ethnic population as a major feature of Malaysian nationhood, there is little evidence of social interaction across ethnic groups and the community engagement process had to take account of the entry points for each of the ethnic groups.

Segamat was selected following a feasibility study that was undertaken in 2010 to explore the establishment of the platform and to trial various data collection techniques. The criteria for the selection of the location included that the ethnic mix of the population approximated that national levels; the population was relatively stable to support a longitudinal study; we could obtain local government permission to establish the platform; and that the population were receptive to the partnership. A further advantage of Segamat was that the Monash School of Medicine had established a prior relationship with the district health authorities for the placement of the medical students to undertake their rural health training in the district. There was therefore a pre-existing relationship and mutual trust on which a further research relationship could be built. The community engagement process started approximately a year before the launch of the platform and is an ongoing process.

## Methods

An instrumental case study approach (18) was taken in order to investigate the implementation and outcomes of the community engagement strategy. An instrumental case study design focuses on the nature and effects of a phenomenon – so although the bounded systems or ‘variables’ need to be defined, these systems are recognized as being complex, dynamic and interrelated and therefore require multiple methods and sources of data (19). Essentially the case study design recognizes the dynamic nature of ‘real-life’ research making it possible to document and continuously analyze the iterative and necessarily flexible processes involved in community engagement and the context in which these processes evolve.

In order to define the boundaries of the case study, we define community engagement as the process of working collaboratively with and through groups of people affiliated by geographic proximity and/or special interest, to address issues affecting the research priorities for their health and well-being (15). The process involves various levels of participation, empowerment, and capacity (20).

The most concrete outcome for us, of a successful community engagement process for the SEACO platform, was the recruitment and retention of at least 80% of the population (21–23) within the selected *mukim*. However, other key objectives were to establish effective structures for communication that would keep the community informed; establish sustainable consultation processes that would enable input from the community about their research priorities in the long-term; and empower communities through the use of the SEACO Platform to promote self-directed change and advocacy. These indicators were relevant for each of the communities within SEACO.

### Identifying key stakeholders and key informants

For the purposes of the community engagement strategy, we placed the SEACO community into two broad categories: communities of interest and communities of place. Communities of interest are the social and political interacting units with interests in SEACO such as local government and the scientific community. Communities of place encompass families and groups of people, such as the social clubs, religious groups and so on, living and interacting within the geographic boundaries of the five sub districts or *mukim* of Segamat selected for SEACO.

Engaging with government was a critical first step to the conceptualization of SEACO. There is a general deference to authority in Malaysia (24) and SEACO would not have been possible without strong tangible support from government. The public services adhere to implicit hierarchical protocols, the observance of which is critical to facilitate access to information and support. It was not possible for instance to obtain maps of municipal areas without the written permission from the district office, which in turn required permission from the state government. Social and political networks had to be carefully mapped out in order to gain a clear sense both of who the key stakeholders were and the order in which they needed to be approached. SEACO therefore has representation from the Federal and State Ministry of Health in its governance structures and works directly with the district health services, and local government offices.

A further aspect to this process was learning how to use the authority that was gained through having ‘high level’ support. The legitimacy provided by government permission had to be constantly negotiated because of the potential for misinterpretation; and where in some instances it was important to tangibly wield government support, in others it was regarded either as a threat or a tool for coercion. At one community meeting for instance, a community representative wanted to know whether SEACO was a backhanded way for the government to pry into their personal matters; but for others endorsement by the state and federal departments gave the legitimacy to demonstrate that we had the right links and therefore the potential to move the findings of the research outside purely academic interests to practical policy application.

Data collection commenced with rapid assessment procedures (25) as part of an ongoing ethnographic study of the people and the district of Segamat. Specific research tools for the case study included detailed field notes (26) from participant observation in community meetings, focus groups with key community groups and in-depth interviews of key informants within the community. Field notes were supplemented with the use of *Bugify* (27), an issue-tracking program. Issue tracking systems are software packages that allow ‘tickets’ to be created to highlight a problem, and within the organization, the ticket remains open until the problem is resolved. Members of the team with shared responsibility for the issue can update the ticket based on various solutions, ensuring an ongoing record of the processes followed toward resolution. Over time, a database of the processes is created with critical institutional memory explaining the reasons for procedures and decisions. *Bugify* provided us with an ideal tool for a sustained record of problems that arose through the community engagement and the solutions that were developed to address them. Weekly field team meeting provided a further opportunity to discuss, document and update the record.

Interviews were initially unstructured, enabling a grounded theory approach to understanding the context of the community (26, 28). Interview and focus group discussion guides could therefore be developed iteratively allowing new questions that were raised and preliminary interpretations to be presented back to community groups for triangulation and validation.

### The community engagement strategy

The community engagement strategy was based around three key activities: creating the representative structures to enable community consultation and participation; establishing mechanisms for information exchange, particularly for individuals in the community to engage directly with SEACO; and establishing processes for community-directed involvement in SEACO research and activities.

### Community consultation and participation

The community engagement strategy was led initially by three key individuals from the local Segamat community: a retired matron who had been the head of the district nursing services, a retired Ministry of Education official and retired senior health practitioner of the district health services. These key persons were designated the core community engagement team and extended our networks to primary care services, education services and the three ethnic communities. They also provided training to SEACO staff about working with communities at multiple levels.

SEACO is led by Global Public Health in the School of Medicine and Health Sciences at the Monash University campus in Malaysia. Research staff directly involved with SEACO are predominantly expatriate academics from the campus; staff responsible for the management and all the operations at the site are mainly Malaysian. Disciplines covered include social epidemiology, medical anthropology, demography, community medicine, information technology and data base management and statistics. Training for field staff therefore covers data collection techniques for surveys and qualitative techniques.

Led by the core community engagement team, we initiated the local community engagement process through key community networks reflecting government and other political structures, ethnic leaders, leaders within the business communities, NGO and charity organizations, and social clubs such as the Lions’ that support those who for various reasons, fell outside the more formal support structures. These alternative entry points were critical to ensure representation from groups as diverse as the plantation workers and residents in federal land development (FELDA) communities, orphanages, and women's shelters. These different subgroups were identified on the basis of multiple consultations to gain an understanding of social and cultural groupings in the district.

Multiple meetings facilitated by the individuals within these various groups were organized in the first instance to inform these key stakeholders about SEACO and to seek feedback on acceptability of the overall concept of the research platform. Interactive discussions were then held about the processes that were required to foster wide spread participation. Examples of these initial meetings include a meeting of the clan leaders of the Chinese communities; meetings of the district assembly members and village heads of the mukim, neighborhood watch groups, the local constabulary, local primary health care clinics, and local membership of the political parties. These groups provided various suggestions for increasing participation of their communities and stressed the need for assurance of privacy and confidentiality particularly given the use of data collectors from within the community.

The level of active engagement during these meetings varied across individuals and the different groups and there were a number of challenges. It was noted, for instance, that SEACO's focus on health and well-being of the community provided potential political advantage in the lead up to local and national elections. It was therefore critical to manage and avoid the perception of an alignment with any political faction. It also became evident that the location of the meeting and order in which participants were invited was significant for participation. There was a group for instance that boycotted one of the initial community meetings because their representative had not been approached personally and the invitation had come via other social networks. The desire to be included though meant that he conveyed his displeasure, providing us with an opportunity to make amends and observe the appropriate protocols.

These multiple meetings have culminated in a core of five permanent, formally constituted community engagement committees (CECs) – one for each of the SEACO mukim. The formation of the CECs was also led by the core engagement team with the brief from SEACO to encourage inclusiveness and participation from all the sectors. While there were some people the research team thought would be valuable additions to the CECs, the final selection was left to the community members who attended consultations to decide. Having attended these meetings however, the team was able to compile a list of key resource persons who were willing to be consulted in a less formal capacity than as members of the CECs.

The CECs have formalized terms of reference and office bearers and take the responsibility very seriously. The CECs meet every 2 months and exchange information with SEACO staff about pending activities, and any problems or opportunities identified in the community that will enhance the SEACO research. The CECs have played an active role in priming the community for upcoming data collection rounds, and have provided advice to SEACO staff about strategies to enhance participation either by being more selective about the time of day a household is approached, or the most appropriate person to approach within a household. Some CEC members also play a ‘door knocker’ role; they accompany data collectors to particular households when the data collector is not known to the community and therefore is able to provide an introduction. This role is carried out on an ad hoc basis and the more mature data collectors rarely call on this resource. Consent to participate still remains with the household; the CEC member's role in this instance is purely to facilitate the dialogue with the data collector. A further example of a role played by the CEC is the management of negative rumors and misinformation that occurred in one of the villages.

It is important to note as well that the CEC meetings have provided a forum that brings together various sectors of the community that did not previously have occasion to come together. Recent meetings where SEACO staff have presented maps and summary feedback data about local areas have fostered active discussions about issues hitherto unrelated to SEACO's current research priorities, such as enhancing local neighborhood security and working together toward dengue mosquito control. These discussions present the opportunity to record concerns and possible future topics for research that are generated and driven by the community of Segamat.

### Community-directed involvement

An early decision was made to devolve aspects of community engagement to the community members themselves. As part of the early consultation process, community members were asked to volunteer to coordinate activities and events that would bring people together and provide the opportunity for open dialogue about SEACO, its objectives and potential benefits to the community ahead of any data collection. The community-directed events therefore included coloring competitions organized for young children that brought families out to the community centers; dance competitions, tai chi, silat, and other exercise sessions. One of the committees brought together an amateur acting group and scripted and staged a performance that demonstrated what a data collection visit would involve. The various events committees were given a modest budget for each event and committees chose the dates, time and place. For most of these events, SEACO partnered with the district public health team to offer free health screening and health promotion and nutrition consultations and where available, local dignitaries attended to lend their support. Support was also provided to facilitate the attendance of those who did not have easy access to transportation and who otherwise would not attend local community events.

In addition, SEACO staff were invited to any large community events that were organized by established NGOs and social clubs including the Lions’ club and the breast cancer support society. SEACO staff were and continue to be visibly present and participate in community activities such as dengue community awareness programs and community clean-ups.

### ‘Marketing’ SEACO in the community

Engaging the community required providing them with information about who we are and our vision and mission. This was done through a combination of media programs and building relationships with key media figures within the local community. In addition to national newspapers, there is a range of local tabloid newspapers available to each of the three ethnic groups. Appropriately targeted pieces were distributed to each of the newspapers and contact journalists are invited to regular community briefings presented by the SEACO staff.

A logo was designed for SEACO that incorporated shadow drawings of mother, father and child into a hibiscus, the national flower of Malaysia. The branding was made visible on banners and buntings in key areas across the district: at intersections, outside clinics, schools and community centers and on the uniforms of all the staff and community members associated with the research platform. The by-line adopted was ‘research for a healthy community’. Information leaflets were made available in Malay, Chinese, Tamil and English and distributed to popular cafes and eating locations.

Key contacts made in the communities, some of who are now members of the CECs, and others who have been formally trained and continue to work as community data collectors, helped with the dissemination of information to the communities and became recognized as informal contacts where community members needed clarification about participation. The contacts referred queries back to SEACO field supervisors.

A further medium that was used was the local community social networking site which provides information on various SEACO activities, notices for employment vacancies with cross links to the SEACO website. SEACO staff also use Facebook to engage with the Segamat community. The SEACO website features web pages targeted specifically at the Segamat community and significant events and cultural and religious festivals are also featured on the regularly updated home pages of the web site. A promotional video was produced featuring community members explaining their understanding of SEACO and the potential benefits to the community. The video is available through the SEACO website in the local languages. Feedback received is followed up by the SEACO field team and catalogued for ongoing analysis.

## Results

The community engagement activities started about 3 months ahead of the official launch of the platform in November 2011. Data collection for the census round did not begin until March 2012. Data collection was preceded by a flyer drop where information sheets about SEACO and the census round were distributed to each household within a week of when the data collector was intending to visit. Therefore, by the time the visit was made for data collection, most households had some idea about the research platform. The initial census and enrolment was undertaken over a period of 9 months working with one *mukim* at a time and covering a total area of about 1,250 km^2^. The initial census included demographic and socio-economic items, and some basic health, and health service use questions.

A strong preference for local data collectors was expressed through community meetings. We therefore managed to recruit a casual workforce of approximately 65 data collectors from the community comprising a combination of retired professionals such as nurses, teachers and civil servants, mothers whose children had left home and significant numbers of younger university graduates and school leavers. The turnover of data collectors has been high particularly in the younger ages. However feedback from the CECs suggests that the opportunity for training provided for data collectors was valuable. Retention of a regular data collection workforce however is a challenge that needs to be addressed.

In the census round, we succeeded in enrolling approximately 85% of the total population of the five selected *mukim* – a total of over 40,000 people. In some villages, the response rate was 100% but for the most part varied between 70 and 95%. In the lowest response *kampong* (hamlet), we achieved a response rate in the census of just over 40%. The reason given for this low rate by the relevant CEC was the timing of SEACO data collection which occurred just after security related incidents associated with the elections. Residents were reluctant to answer the door to strangers in spite of the SEACO uniform. However, the response rate in that Kampong improved in the subsequent round of data collection conducted 9 months later, to update the census information.

As part of the operations quality control process, data collection supervisors are required to make random visits to households that had been visited by data collectors, in some cases to verify the data and in others just to check that there had indeed been a visit by a SEACO data collector. Supervisors visited all households where there was an initial refusal to participate to inquire about the reason for refusal. In many cases the reason given was that the household had a preference for a data collector from the same ethnic group and in those instances, it was possible to convert the refusal into an enrolment. For other refusals, reasons included that they were uncertain they wanted to participate but were open to being approached at another time. A number of these actively sought out data collectors to return and interview their families after they had conferred with neighbors who had been interviewed.

As part of an established operations procedure, data collectors produced local area maps of their assigned areas with color codes of completed households, absolute refusals and houses that appeared to be unoccupied. These maps demonstrated that there were patterns to acceptances and refusals. There was clear clustering of households that did want to be enrolled. Refusals were also unlikely to occur in homes around those of data collectors, CEC members and areas close to community centers or areas in which there was clear SEACO advertising. This highlights the importance of sensitization in the community engagement process and informs the SEACO operations about where effort might be put to enhance recruitment. Furthermore, this provides critical data for understanding and interpreting biases in the analysis of the data.

Finally as part of the information exchange activities, community briefs are produced for each mukim at the end of a data collection round. The briefs are available through the district office and the local newspapers provide regular summaries of the profile of various communities as the results become available. Presentations are made to community members through events organized by the CECs. The last of these meetings resulted in a community led initiative to begin a project on safety within their local area. While ultimately, this initiative did not require the involvement of SEACO staff, the role played by the SEACO CECs in bringing the community members together and the data provided to drive this project is a promising sign of the development of a sustainable partnership with the community of Segamat. The new safety initiative will be monitored as part of the community engagement case study.

## Discussion and conclusion

An extensive and ongoing process of engagement was undertaken in the SEACO research community. The purpose was not only to facilitate recruitment, retention, and quality of the data collected, but also to help ensure the interests and well-being of the community, and ultimately to have the research platform become a vehicle for community-driven research. The community engagement process required a rich and nuanced understanding of the context in which the SEACO platform was to be established.

A critical lesson from the instrumental case study findings is the importance of relationships in the recruitment process. A recruitment rate of 85% likely represents a proxy for the success of the community engagement process. This success can mainly be attributed to the initial identification of key social and political stakeholders; involving the community in engagement and relationship-maintenance strategies; and ongoing ‘marketing’ of the research to increase awareness and foster familiarity in the community. Government support at the local and national level has been crucial to this process, as have the community-lead engagement strategies via informal (community events) and more formalized structures such as local CECs. We did however need to be constantly analytical about the processes and the sources of information from the various stakeholders, as well as the relevance of information to different sectors of the community.

While the importance of strong relationships is well known in anthropological research (29, 30), the implications are often ignored in large population based studies. Indeed concern is often expressed about the impact and therefore lack of generalizability of results if the population is affected by the design of the research (21). Notwithstanding implications for recruitment rates, retention and data quality in longitudinal, population-based studies, we believe that ignoring these relationships presents a missed opportunity to systematically analyze the changes that may indeed be brought about by the ‘intervention’ of establishing an HDSS site.

Since the official launch of the SEACO platform, there have been a number of ongoing studies. An ethnographic study is underway to provide an understanding of the cultural context of the people in the district. There is also a qualitative study to explore health related behaviors in adolescents across the three ethnic groups. A small project has been established to explore the quality of life of elderly men and women living on their own and similar studies are focusing on patients with stroke and households with diabetics. From the operations perspective, procedures have begun to explore ISO certification for quality management processes. It is therefore critical that an ethos of self-evaluation and reflection is introduced as fundamental to all aspects of the work of the platform.

Case study research is necessarily limited by the intensive focus on the single case. However, the particular feature of a case study is the opportunity to explore the nature of complex processes and through that exploration, draw out relevant lessons that allow a better understanding under different contexts. The instrumental case study approach, which is now embedded as part of the SEACO operations, provides the opportunity for an ongoing record of a research process and community in evolution and the impact of this on research and research findings. While the context of every population-based cohort study or HDSS will necessarily differ, our approach to studying the implementation and likely outcomes of the community engagement strategies undertaken in SEACO, may help inform such processes for similar studies and sites elsewhere.
